# Inelastic Scattering of H Atoms from Surfaces

**DOI:** 10.1021/acs.jpca.1c00361

**Published:** 2021-03-29

**Authors:** Oliver Bünermann, Alexander Kandratsenka, Alec M. Wodtke

**Affiliations:** † Institute for Physical Chemistry, Georg-August-University of Göttingen, Tammannstrasse 6, 37077 Göttingen, Germany; ‡ Department of Dynamics at Surfaces, Max-Planck Institute for Biophysical Chemistry, Am Faßberg 11, 37077 Göttingen, Germany; § International Center for Advanced Studies of Energy Conversion, Georg-August University of Göttingen, Tammannstrasse 6, 37077 Göttingen, Germany

## Abstract

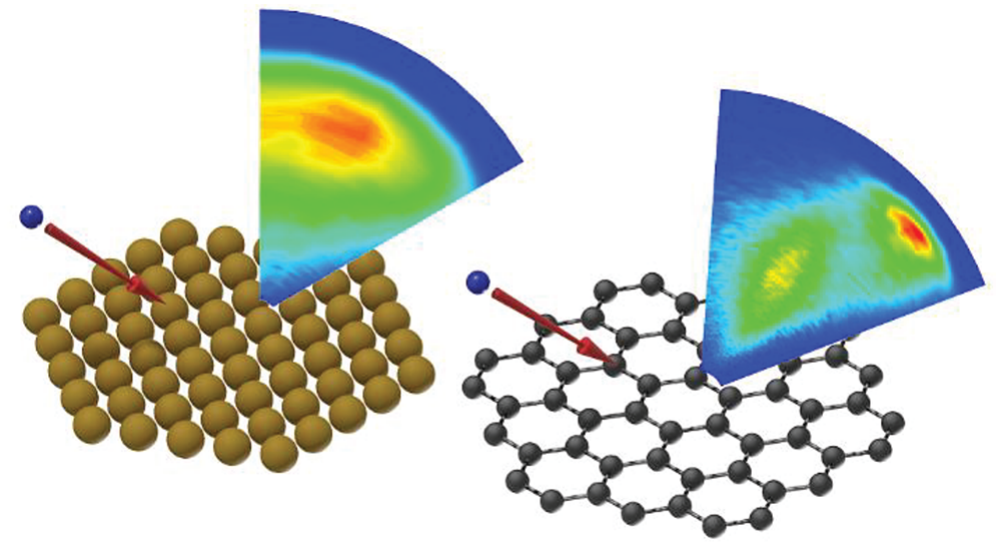

We have developed an instrument that uses photolysis of hydrogen halides to produce nearly monoenergetic hydrogen atom beams and Rydberg atom tagging to obtain accurate angle-resolved time-of-flight distributions of atoms scattered from surfaces. The surfaces are prepared under strict ultrahigh vacuum conditions. Data from these experiments can provide excellent benchmarks for theory, from which it is possible to obtain an atomic scale understanding of the underlying dynamical processes governing H atom adsorption. In this way, the mechanism of adsorption on metals is revealed, showing a penetration–resurfacing mechanism that relies on electronic excitation of the metal by the H atom to succeed. Contrasting this, when H atoms collide at graphene surfaces, the dynamics of bond formation involving at least four carbon atoms govern adsorption. Future perspectives of H atom scattering from surfaces are also outlined.

## 1 Introduction

Since the discovery of quantum mechanics nearly 100 years ago, the underlying physical laws governing chemistry have been known, but the computational implementation of those laws has remained completely intractable. Michael Polanyi and Henry Eyring understood already in 1931 that theoretical chemistry is all about approximations^1,2^ and one of the great achievements of modern theoretical chemistry is to show how suitable approximations may be exploited to make quantitative predictions of the observable properties of simple gas-phase chemical reactions. Perhaps the best example is the H + H_2_ → H_2_ + H reaction. While Eyring solved Newton’s classical equations of motion using a semiempirical potential energy surface for three reacting H atoms constrained to move on a line, only in 1958, using a digital computer with 2800 vacuum tubes and weighing five tons, could this be done for enough trajectories to learn about the reaction.^3,4^ With all the approximations needed, it was essential to test the developing theoretical methods, and consequently, new experiments were invented and developed. Conventional crossed molecular beam methods were applied^5−10^ and then improved with the use of photolytic atom sources.^11−14^ The development of resonance enhanced multiphoton ionization (REMPI)^15−19^ led to field free ion time-of-flight (TOF), Rydberg atom tagging detection^20−36^ and ion imaging^37−39^ eventually enabling measurement of the differential scattering cross sections for selected quantum states of the reaction products with controlled incidence translational energy. As experiments improved, so did theory, and by the end of the last century, the technology was sufficiently developed so that converged quantum dynamics calculations on an accurate Born–Oppenheimer potential energy surface (PES) could be achieved.^40,41^ Eventually, it was possible to demonstrate quantitative agreement between theory and experiment at an extraordinary level of detail. This led to observations of interference between theoretically predicted^41^ quantum bottleneck states^33^ and clear evidence that the geometrical phase effect^36,37^ influences H + H_2_ → H_2_ + H. It is now indisputable that gas-phase chemical reactivity is nothing more than multidimensional quantum motion of nuclei on a Born–Oppenheimer PES, one that can often be calculated accurately by modern electronic structure theory. This has been called the standard model of chemical reactivity.^42^ Extending the standard model to account for hopping between multiple PESs has also been achieved.^43^

Coming to the subject of this review, we do not currently know if the theoretical methods used so successfully for understanding the dynamics of gas-phase reactions are valid for surface chemistry. Typical assumptions that work well for gas-phase reactions may not hold for surface reactions, the most fundamental of which is the electronically adiabatic approximation of Born and Oppenheimer (BOA).^44^ Hence, one focus of the current research is to examine electronically nonadiabatic processes in chemical reactions on surfaces.^45−50^ Numerous experiments report observations that indicate the coupling of nuclear and electronic degrees of freedom.^51−57^ Reactions of open-shell molecules on low work function metals can lead to surface chemiluminescence,^58^ emission of exoelectrons,^59^ or negative ions.^60^ Vibrational relaxation lifetimes of molecules, chemisorbed^61,62^ or physisorbed^63^ on metal surfaces, are typically between 1 and 100 ps. On the other hand, electronically adiabatic vibrational energy transfer to phonons can require many milliseconds.^64,65^ Furthermore, when a molecule scatters in a subpicosecond collision from a metal surface, its vibrational motion can be thermally excited, a process that does not occur on insulator surfaces,^53,66,67^ and when highly vibrationally excited molecules collide with low work function surfaces, electrons can be emitted.^54^ Applying Schottky diodes^51,52,68−70^ or metal–insulator–metal contacts^71,72^ as detectors for hot electrons created in a surface reaction provides a direct albeit qualitative measure of electronic nonadiabaticity. These examples strongly suggest that electronically nonadiabatic effects are important in chemical reactions at metal surfaces. Unfortunately, it is difficult to find experimental data capable of accurately quantifying the electronic coupling, information that is necessary to provide benchmarks to challenge theory.

When constructing theoretical models for surface dynamics and chemistry, there are two main approaches to account for electronically nonadiabatic effects: the so-called electronic friction (EF)^47,73,74^ and independent-electron surface hopping (IESH).^75,76^ In molecular dynamics with EF, nuclei move on the ground-state PES and experience the interaction with electron–hole pairs (EHPs) as a drag force. IESH, on the contrary, considers explicitly the time evolution of many electronic states with the Schrödinger equation, and nuclear motion occurs on a PES. The former method has been successively applied to model the coupling of the translational degrees of freedom of adsorbates at metal surfaces,^77^ whereas the latter approach was successful in understanding the energy exchange between EHPs vibrational degrees of freedom of adsorbates.^78^

In addition to the Born–Oppenheimer approximation, it is also important to test the classical approximation as the number of atoms involved in surface reactions typically prevents the use of quantum theories of nuclear motion; hence, nuclear quantum effects like zero-point energy (ZPE), tunneling, or quantum resonances are often neglected. We know from gas-phase studies that these effects can have a profound influence on chemical reaction dynamics,^79−82^ especially if light atoms like hydrogen are involved.^83−86^ The classical approximation also simplifies the treatment of a solid’s phonons. The validity of the classical approximation is also unknown. Questions like these have stimulated the invention and development of new quantum dynamics theories, including reduced dimensionality quantum wave packet calculations,^87^ ring polymer molecular dynamics (RPMD),^88−91^ and multiconfiguration time dependent Hartree (MCTDH) methods.^92,93^ However, careful comparisons of theory and experiment to test these theoretical approaches are still limited.

To validate new theoretical methods, high-level benchmark experiments under well-defined conditions with sufficient experimental resolution are required. A common strategy is to pick one of the elementary steps of the process and study it on a well-defined model system, an approach suggested by Langmuir as early as 1927^94^ and pioneered by Ertl.^95−97^ Experiments on the inelastic scattering of atoms and molecules from well-defined surfaces under ultrahigh vacuum conditions allow us to probe the mechanisms of dissipation in great detail, addressing fundamental questions related to adsorption,^98^ desorption,^99^ diffusion, and reactivity;^100^ in short, these are all of the elementary steps needed for surface chemistry to take place. Based on such experiments, new theoretical models can be developed and tested that accurately describe the delicate interplay between electronic and nuclear motion in prototypical chemical reactions, a capability that is necessary for accurate predictions of reaction rates in heterogeneous catalysis.

Theoretical predictions of reaction rates can be nearly exact using Transition State Theory (TST), if a dynamical recrossing of the transition state is either included in the rate calculation, known from experiment, or unimportant in the system being studied. Adsorption and desorption are particularly interesting in this regard, as it can be shown that the sticking coefficient, *P*_*S*_, can be used to account for dynamical recrossing.^101^ Specifically, the rate of adsorption, *R*_*ads*_, is equal to the equilibrium one-way flux through the transition state, *F*_*TST*_, reduced by *P*_*S*_. In addition, the principle of detailed balance requires that the equilibrium adsorption rate be equal to the desorption rate. These statements are concisely reformulated in eq 1.
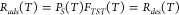
1This equation shows that the nonequilibrium influence of dynamics on the rates of adsorption and desorption is encoded within the sticking probability’s dependence on incidence energy and everything else that it may depend on, e.g., coverage, surface temperature, incidence quantum numbers, and so forth.

Accurate and detailed measurements of inelastic scattering with surfaces can provide the foundation for validated theories of adsorption and—because of detailed balance—desorption. Such measurements therefore become an extremely valuable testing ground for understanding dynamical influences on surface reaction rates as their comparison can shed light on the validity of the Born–Oppenheimer and classical approximations, as well as other assumptions that might form the basis of an approximate theory.

Previous inelastic scattering experiments focused on molecules, which add an additional level of complexity—molecular rotation and vibration can couple to surface excitations and with each other. Early experiments focused on the rotational excitations of the scattered molecules, e.g., observing rotational rainbows.^102−104^ Later, the coupling of the molecular vibration to EHP excitations became of central interest.^53,54,66,67^ Today, the experiments have improved so much that we can control and determine all degrees of freedom including translation simultaneously. Here, the theory still struggles to give the correct description,^105^ and it would be highly desirable to take a step back and do experiments on simpler systems, specifically atoms, where we focus on the translational degrees of freedom. Rare-gas atom surface scattering can be used to obtain information on surface phonons^106−108^ and to distinguish direct scattering from trapping–desorption.^109−112^ Unfortunately, rare gas atoms tell us little about surface chemistry.

Hydrogen is the simplest open-shell atom, and understanding its surface dynamics has implications ranging from interstellar chemistry^113−118^ to maximizing the performance of neutral beam injectors at the International Thermonuclear Experimental Reactor (ITER).^119^ Due to its simplicity, H atom surface scattering is particularly attractive to make detailed comparisons between experiment and first-principles theories.^77,120−122^ Furthermore, due to its low mass, an electronically adiabatic picture predicts inefficient energy transfer to most solids. Hence, H atom interactions with solids can be particularly sensitive to failure of the electronically adiabatic approximation. Furthermore, hydrogen is an ideal candidate to test the influence of nuclear quantum effects and the validity of the classical approximation for nuclear motion.

Inelastic H atom scattering has been limited by poor H atom sources that rarely provide narrow velocity distributions; previously, discharge-based H atom sources with electromagnetic velocity filters were used.^123,124^ Detection of H atoms is also challenging: Bolometers,^124^ ZnO conductivity detectors,^125^ and photographic plates^126^ are sensitive to H atoms, but their slow temporal response has restricted all previous experiments to spatially resolved diffraction measurements.^126−130^

Inspired by work in gas phase chemical dynamics, we have recently developed a new experimental tool to study inelastic H atom scattering from solid surfaces.^131^ The apparatus combines Rydberg atom tagging^21,23−35^ with photolytic H atom beams using hydrogen halides as precursors,^300−167^ in a design that is compatible with ultrahigh vacuum surface scattering. Our new apparatus provides scattering energy and angular distributions with variable incidence energies ranging from 200 meV to 7 eV and energy widths as narrow as 2 meV or even narrower. This paper reviews key findings achieved with this instrument, emphasizing those that have benefitted from a fruitful interplay with theory. One focus is the interaction of H atoms with metal surfaces. Here, we find that electron–hole pair excitation of the surface explains the high adsorption probabilities for H atoms on metal surfaces.^132^ The second focus is the interaction of H atom with graphene, a surface where covalent bond formation is possible. Here, we find an extraordinarily rapid energy transfer process exciting graphene’s phonons that is induced by the electronic rehybridization of the carbon atom involved in the transient C–H bond formation.^133^ The paper concludes with a section describing new ideas and future possible research directions.

## 2 Experimental Methods

**Figure 1:**
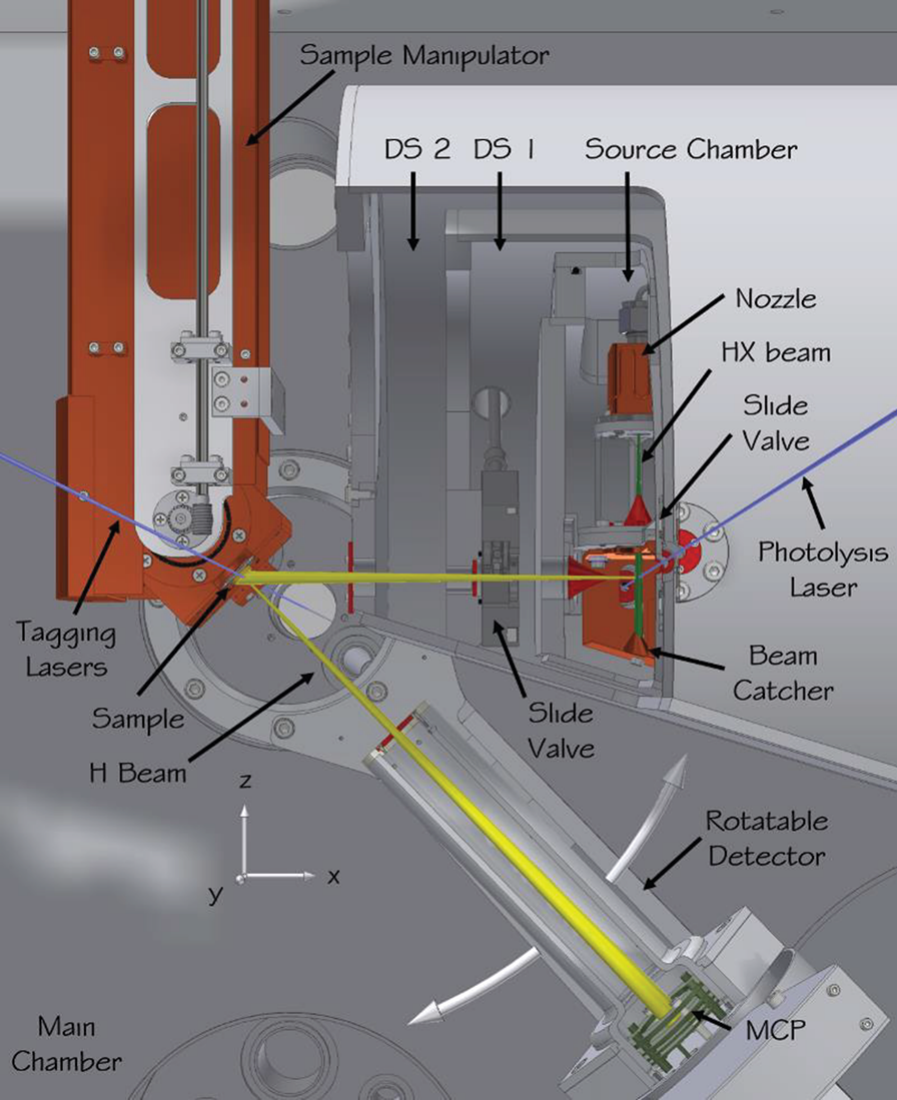
Apparatus for scattering with photolytic H atom source and Rydberg atom tagging detection. In the source chamber, the hydrogen halide molecular beam (green) is formed in a supersonic expansion from a pulsed nozzle, passes a skimmer (red), and is intersected by the photolysis laser (blue) before it hits a liquid nitrogen cooled beam catcher. Part of the generated H atoms (yellow) leave the source chamber through a second skimmer, pass two differential pumping stages, and enter the main chamber where they collide with the sample surface. The sample is mounted on a six-axis manipulator allowing the incidence polar and azimuthal angles to be varied. The surface temperature can be cooled to ∼45 K using a flow cryostat and heated to ∼1500 K using an electron bombardment heater. The scattered H atoms are excited to a metastable Rydberg state by the tagging lasers, pass an aperture defining the angular resolution of the detector, and, after a 250 mm flight path, are field ionized, and the ions are detected by a MCP detector. The detector is mounted on a rotatable arm to enable variation of the scattering angle. A coordinate system is included to define the *x*, *y*, and *z* axes.

Figure 1 shows key components of the apparatus, which has been described in detail elsewhere.^131^ The vacuum system consists of a source chamber, two differential pumping stages (DS 1 and DS 2), the main scattering chamber, and a sample preparation chamber (not shown), to which the sample can be moved by translating the manipulator along the +*z* direction. The source chamber is pumped by a cryopump (COOLVAC 1500 CL-V, Oerlikon Leybold Vacuum GmbH) and houses a pulsed nozzle to generate a supersonic beam of hydrogen halide (HX) molecules (green), which is skimmed (red) and condensed on a LN_2_ cooled beam catcher (copper). DS 1 and 2 are pumped by turbo molecular pumps (TMPs) (HiPace 300 M, Pfeiffer Vacuum GmbH) and lower the gas load on the scattering chamber. The main chamber is equipped with a 2000 L/s TMP (ATP 2300 M, Pfeiffer Vacuum GmbH) and copper shields in the line of sight of the sample surface that can be cooled to LN_2_ temperature. A pulsed UV laser beam (blue) crosses the molecular beam (green), photolyzing HX.^300−167^ A small fraction of the resulting H (or D) atoms (yellow) pass a second skimmer (red), separating the source chamber from DS 1, an aperture separating DS 1 from DS 2, and a second aperture separating DS 2 from the main chamber—both apertures are shown in red. The atoms then enter the main chamber, where scattering from the solid sample (gold) takes place. The scattered atoms are detected by Rydberg-atom tagging,^20−35^ where the atoms are first excited via their 1s–2p transition at 121.57 nm and subsequently at 365 nm from the 2p state to a Rydberg state just a few cm^–1^ below the ionization limit, typically *n* = 30–70. These Rydberg atoms are metastable and travel 250 mm without radiative loss to a rotatable detector, where they are field-ionized. A microchannel plate assembly then amplifies each ion, and a multichannel scaler records the TOF with respect to the synchronized tagging laser pulses. The detector is mounted on a rotatable arm with one aperture, shown in red, providing selection of the scattering angle *ϑ*_s_ with a 3° angular resolution.

The geometry of the vacuum chambers involves embedding the source region within the two differential regions, something like a Russian matryoshka doll. Consequently, the photolysis laser beam enters from the apparatus through a transparent vacuum seal into the main chamber and passes through an aperture separating the main chamber from DS 2 and then through a second aperture separating DS 2 from DS 1 before passing through a third aperture separating DS 1 from the source chamber. Similar apertures are present allowing the laser beam to exit the apparatus. Slide valves with O-rings are installed to seal between DS 1 and DS 2 in three places (laser beam entrance and exit as well as behind the skimmer where the H atoms exit the source chamber) so that the source chamber and DS 1 can be vented without disturbing the ultrahigh vacuum (UHV) regions of the instrument.

Several measures are taken to limit contamination of the surface by hydrogen halide molecules and halide atoms. First, the vacuum was improved 1000-fold compared to previous Rydberg atom tagging instruments in order to maintain a clean surface during the long measurement times. This was accomplished with standard UHV techniques including all metal seals, careful choice of high temperature materials and with an automated baking mechanism that is typically used to uniformly heat the chamber to 120 °C for several days. Liquid nitrogen is also used to condense HI in the source chamber. In addition, the cryopump is positioned so that the surface view into the source chamber sees only a cold surface within the cryopump. This prevents HI from directly flying from the source chamber to the surface.

A six-axis manipulator was constructed in our shops and installed in the scattering chamber to position the solid-sample and control its temperature. The manipulator provides translation in the *x*, *y*, and *z* directions, as well as rotation about the *y*- and *z*-axes and azimuthal rotation about the crystal normal. The manipulator is used to transport the sample (*z*-direction) to a preparation chamber, where surface cleaning can be performed with argon ion sputtering and where low energy electron diffraction (LEED) and Auger electron spectroscopy (AES) are used for sample characterization. A hydrogen atom cannon and an UHV leak valve are also present here for surface dosing. We recently added a load lock for rapid exchange of samples. Rotation about the *z*-axis allows for positioning the sample in front of various devices in the preparation chamber; rotation about the *y*-axis provides control of the incidence polar angle *ϑ*_in_ and azimuthal rotation about the crystal normal of the azimuthal incidence angle *φ*_in_. The sample temperature *T*_S_ can be adjusted between 45 and 1500 K, employing electron bombardment heating and flow cryostat cooling with liquid nitrogen or liquid helium.

Four light sources are available for photolysis. KrF (248 nm) and ArF (193 nm) excimer laser radiation produced by a Lambda Physics COMPexPro 205 F with unstable resonator may be used to photolyze HBr, DBr, HI, or DI. This allows experiments with H and D atoms whose kinetic energy spread is Δ*E* ∼ 20 meV. Alternatively, a frequency doubled or tripled nanosecond-pulsed dye laser (Cobra-Stetch, Sirah, pumped by Quantaray Pro-290 Nd:Yag Laser, Spectra Physics) may be used for photolysis, producing H or D atom beams with Δ*E* ∼ 2 meV. In yet another experimental geometry, the tripled dye laser beam (212.5 nm) is mixed with a visible laser pulse from a second dye laser (tunable around 800 nm) to produce tunable vacuum ultraviolet pulses at λ ∼ 120 nm by 2ω_1_ – ω_2_ resonance enhanced four-wave mixing (FWM).^134^

**Figure 2:**
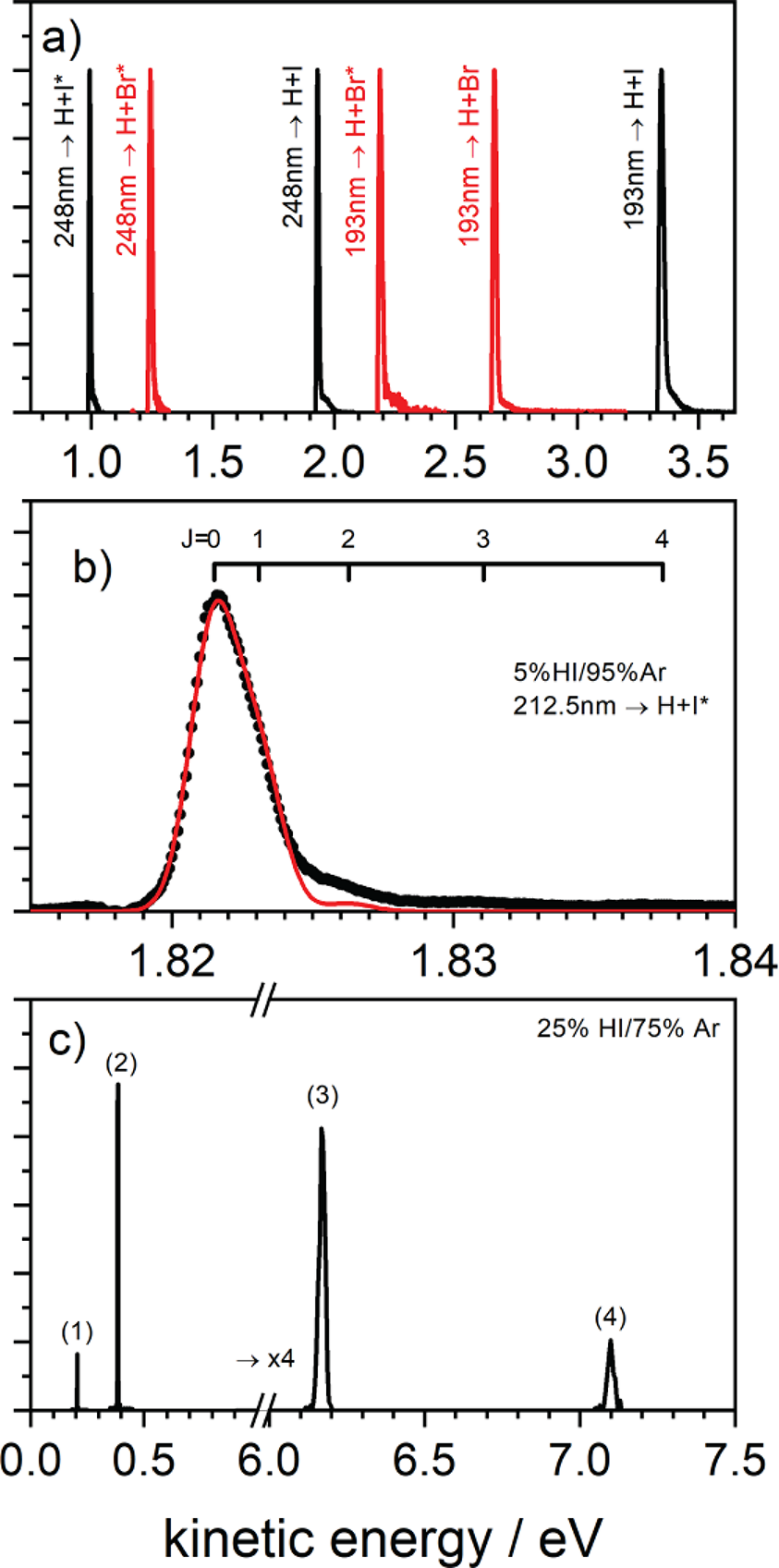
Photolytic H atom beams and their kinetic energy distributions. Panel a shows the kinetic energy distributions of H atom beams formed by the dissociation of HI and HBr with ArF or KrF excimer lasers. Panel b shows the kinetic energy distribution of an H atom beam generated by dissociation of an Ar-seeded HI beam with a dye laser at 212.5 nm. The residual width of the energy distribution is primarily due to excited rotational states populated in the HI precursor. The red solid line represents a fit to the kinetic energy distribution giving an HI rotational temperature of 11 K and a fwhm of 2.7 meV. Panel c shows the kinetic energy distributions of four H beams generated by photolysis of HI with VUV photons: (1) 82406.8 cm^–1^ → H + I*(5p^43^P_0_)^2^[2]_3/2_; (2) 82406.8 cm^–1^ → H + I*(5p^43^P_0_)^2^[2]_5/2_; (3) 82367.2 cm^–1^ → H + I*(5p^52^P_3/2_^o^); (4) 82334.3 cm^–1^ → H + I(5p^52^P_1/2_^o^). Note that the width of beam 1 in part c is narrower than the experimental resolution (<1 meV).

A wide range of incidence translational energies E_in_ may be produced—see Figure 2. At all photolysis wavelengths, halogen atoms are produced in both their ^2^P_3/2_ and ^2^P_1/2_ states. With VUV photolysis, additional states may be accessed.^168^Figure 2a shows H atom beams with energies between 1 and 3.5 eV formed with excimer laser photolysis. Figure 2b shows an example using the tripled dye laser, where the energy resolution is emphasized. Figure 2c shows H atom beams produced with VUV photolysis using FWM. Tunable incidence energies as high 7.1 eV and as low as 0.2 eV can be produced. We call special attention to the H atom beam at 0.2 eV. These atoms are produced by resonant excitation to a single rotational level of a long-lived HI Rydberg state; hence, the energy spread is not limited by the bandwidths of the lasers and is, in fact, narrower than the energy resolution of Rydberg atom tagging detection (∼0.001 eV).

## 3 Theoretical Methods

Experimental data obtained with this instrument are frequently compared to dynamical simulations. The most important prerequisite for a reliable model of the energy exchange between adsorbate and surface is a recipe for both accurate and efficient calculation of the energy of the system in a given electronic state. For periodic systems, this is provided by the density functional theory (DFT) with generalized gradient approximation (GGA) functionals.^135^ Despite the significant increase in the performance of computers in the past decades, the computational power is not sufficient to simulate recent scattering experiments by calculating the energy and forces “on-the-fly” for each nuclear geometry along a trajectory, for it is necessary to run millions of trajectories to accumulate sufficiently small statistical uncertainty.^132,136^ The well-known workaround for that is to fit an appropriate analytical function (or an effective algorithm) representing the potential energy surface (PES) to a set of DFT data obtained for nuclear geometries sampling relevant regions of system’s configuration space.^135^

We found that for H atom at a metal surface, the Effective Medium Theory (EMT) developed by Nørskov and collaborators^137,138^ provides a PES which, on one hand, accounts for a lot of physical properties of metals and, on the other hand, has a reasonably small number of fitting parameters (14 for a H-metal system).^77,120^ The key quantity here is the background electron density experienced by each nuclei, which is the sum of contributions due to all the other atoms. These contributions are calculated by mapping the local surrounding of a nuclei onto a reference system, like a perfect crystal, for which the analytical expressions can be obtained. The EMT-PES is very computationally effective; moreover, it provides “for free” means to account for the nonadiabatic effects, since the EMT energy is expressed in terms of the background electronic density. Disadvantages of EMT-PES consist in its applicability only to modeling interactions between a hydrogen and *fcc*-metals and the fact that it is often not a very accurate fit to the DFT data—100–200 meV RMSE is typical.^77,139^

Recent advances in the application of neural networks (NN) to the chemical systems^140−143^ provide another tool to construct a PES for H atom at a surface with much higher fitting accuracy^144^ —typical RMSE values of less than 10 meV are obtained—than any known analytical function. Unfortunately, the amount of DFT data necessary to train a NN is much larger than that necessary for fitting the EMT-PES; therefore, training can itself be nontrivial. Below we show high-dimensional NN-PES (HDNN-PES) for an H atom at free-standing graphene^145^ using RuNNer.^141,146,147^

With a good PES in hand, one then has to select a propagation scheme for the nuclei. If electronic adiabaticity can be assumed (for example, in case of hydrogen scattering from insulators), Born–Oppenheimer molecular dynamics (BOMD) is the method of choice.^120−122^ It can also be used to answer the question of validity of the Born–Oppenheimer approximation when comparing the results of simulations with the experimental data. To simulate electronically nonadiabatic surface dynamics, one of the simplest ways is based on an idea stemming from the studies of stopping power of ions in metals.^148,149^ Here, the projectile loses its energy through interactions with EHPs of the metal in a way that is analogous to that of heavy particles in a liquid. It feels a systematic drag force and a random force due to the thermal fluctuations, which drive the projectile to the equilibrium with the surface. The drag force is proportional to the projectile’s velocity using a “friction coefficient” as a proportionality constant. With certain simplifying assumptions, the friction is a function only of the background electronic density in the metal.^150^ As the electronic density in metals is spatially inhomogeneous, the “electronic friction” coefficient varies with position. With this model, the Langevin equation can be used to propagate nuclei with the random force determined by the friction coefficient and temperature from the fluctuation–dissipation theorem. This approach is just as computationally effective as the standard molecular dynamics (MD) as long as there is a simple procedure to get the background electron density as a function of nuclear positions. The EMT-PES provides this as well as the energy without additional computational costs.^77^

An additional important issue to address considering the dynamics and adsorption of a light atom like hydrogen at the surface is how large the quantum-mechanical effects are. Ring Polymer Molecular Dynamics^89,151^ provides a means for that, accounting for zero point energy and tunneling effects. Though being approximate and verified only for systems in thermal equilibrium, the RPMD algorithm is computationally efficient, since here the time evolution of the quantum system is represented by some number of replicas of its classical counterpart coupled in such a way that quantum statistics is preserved. The accuracy of RPMD decreases with the simulation time, but on the short-time scales characteristic for the hydrogen motion at surfaces (∼100 fs), one can expect a small accuracy loss.^145^

The PESs and propagation algorithms discussed in this section are implemented into the *md_tian 2* package, written in Fortran and available at the public repository.^152^ All simulations reported in this paper were performed using this code.

## 4 Inelastic Scattering of H-Atoms from Metal Surfaces

H atom adsorption at metals presents an apparent paradox. In order to adsorb, an atom must dissipate its translational energy to the solid, but the binary collision model (BCM) suggests that the H atom’s mass limits its ability to transfer energy to the solid. In case of head-on collision of two hard spheres with masses *m*_1_ and *m*_2_, the BCM predicts an accommodation coefficient for translational inelasticity, α, as shown in eq 2.
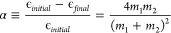
2α goes to zero as the mass of one of the particles becomes small compared to the other. Despite this, experimental observations show that H atoms adsorb easily to metals even at high incidence energies.^153^

Two hypotheses might explain this. The first involves complex scattering behavior involving multiple bounces, penetration of the surface, and/or perpendicular to parallel momentum conversion. The second requires efficient transfer of translational energy to electronic excitations of the metal.^154^ The new H atom scattering apparatus is ideally suited to resolve this and related questions. This required us to also develop a theoretical model to describe H atom interactions with metal surfaces so that theory can be compared to experiment, employing a variety of assumptions about what is important to the dynamics. The close interaction between experiment and theory enables us not only to obtain a detailed understanding of translational inelasticity but also, in addition, to infer an atomic scale view of adsorption.

In the remainder of this section, we first describe the experiments that probe the validity of the Born–Oppenheimer approximation by comparing inelastic H atom scattering for collisions with metals and insulators. We then present results of an inelastic scattering study probing the H/D isotope effect, which allows us to make theoretical connections to chemicurrent measurements. Finally, we present evidence that the behavior found in our limited observations is likely to be universal among other metals, by applying our approach to six different *fcc* metals.

### Mechanism of Adsorption at Metals: Comparing Metals to Insulators

**Figure 3:**
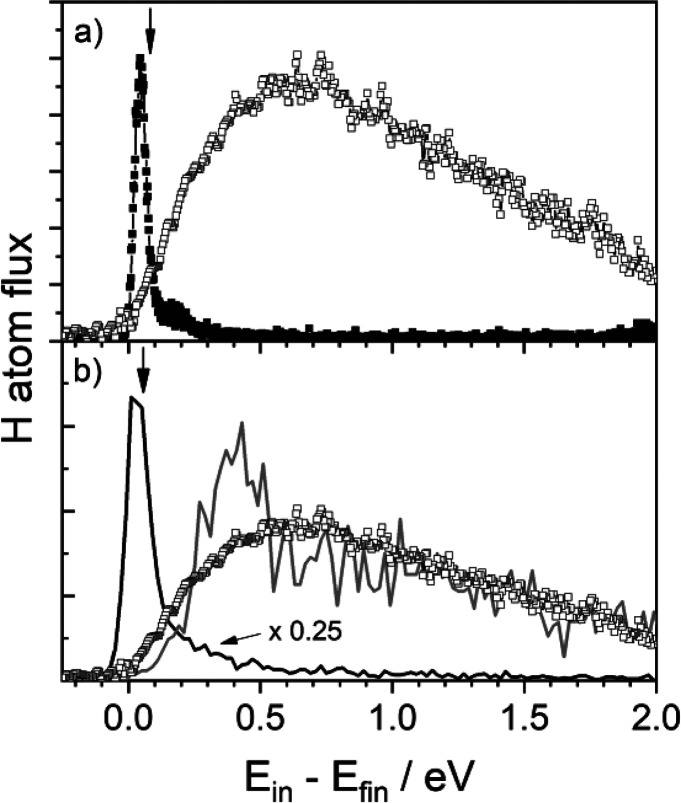
Translational inelasticity for H atom collisions with an insulator and a metal. (a) Measured kinetic energy loss spectra for H atoms scattered from Au(111) (open squares) and solid Xe (filled squares). The vertical arrow marks the expected energy loss for a binary collision between an H and a Xe atom. (b) Theoretical energy loss for H atom scattering from Au(111) found when neglecting (solid black line) and including (solid gray line) electronic friction. The experimental energy loss distribution is shown as open squares. The vertical arrow marks the expected energy loss for a binary collision between an H and a Au atom. Experimental conditions: *E*_in_ = 2.76 eV, *ϑ*_in_ = 45°, *ϑ*_s_ = 45°, and *φ*_in_ = 0°. Adapted with permission from ref (132). Copyright 2015 AAAS.

In demonstrating the coupling of a metal’s electron–hole pairs (EHPs) to molecular vibration, it has proven useful to compare vibrationally inelastic scattering of molecules in collisions with metals and insulators.^53^ In a similar way, we have compared H atom translational inelasticity in scattering experiments with Au(111) and with solid Xe.^132^ Au(111) was chosen because of its high mass and because of its limited reactivity.^155^ Xe was chosen for its high mass and because it is easy to grow a solid layer of Xe on Au(111) at surface temperatures below 60 K.^156^ This allows scattering experiments on the two solids to be carried out in close succession. In practice, we first cooled the Au(111) surface to ∼ 50 K, exposed it to Xe to build up a thick solid layer, and then performed the scattering experiment. Subsequently, we heated the surface to room temperature and repeated the scattering experiment on the now clean Au surface. The experimental results for H atom scattering from the two samples are shown in Figure 3a. While nearly no H atom energy is lost in scattering from solid Xe, a large energy loss is seen with Au(111). The observed H atom energy loss in the Xe experiment is consistent with BCM (black arrow in Figure 3 a)—obviously, the results seen with Au(111) are not.

These observations strongly suggest that, for the case of Au(111), excitation of the metal’s electrons is responsible for the H atom energy loss. To study this more deeply, we developed a theoretical model^77,120^ involving a full dimensional PES constructed by DFT data with EMT. We then performed molecular dynamics simulations with and without a simple model of electronic excitations, implemented by use of the local-density electronic friction approximation (LDFA).^149,150,157^ The model has no adjustable parameters—the electronic density is directly obtained from EMT. Figure 3b compares the two theoretical simulations with experiment. Without electronic excitation, the model produces energy loss that is similar to BCM, in no way consistent with experiment but qualitatively similar to H atom scattering from Xe. When electronic excitation is included, good agreement with experiment is achieved. The theoretical analysis confirms that electronic excitations are of central importance to H atom adsorption on Au(111).

The theoretical model was then extended to study adsorption. Under the conditions of Figure 3, the simulations predict a sticking probability of ∼55%^158,159^ and predict that the dominant adsorption mechanism proceeds first by surface penetration followed by resurfacing. Furthermore, the proclivity of H atom motion to excite electrons leads to domination of multibounce scattering events, something that is also seen but as a minor channel in scattering from the insulating Xe. So in a sense, both hypotheses in combination are correct.

### Unifying Theory of Scattering with Chemicurrents: Isotope Effect

We also studied the isotope effect.^160^ The predicted isotope effect for energy transfer to lattice vibrations versus electronic excitations is diametrically opposed. The energy transfer to lattice vibrations scales with mass; deuterium should transfer about two times more energy than hydrogen. But, according to electronic friction theory, the transfer to electronic excitation should scale with speed: for the same incidence energy—*m*^–1/2^—H should transfer ∼1.4 times more energy than D. Studying the isotope effect of the energy transfer gives valuable information about the interplay between these two effects.

The isotope effect also provides a means of comparing inelastic scattering with chemicurrent experiments. While the magnitude of the chemicurrent can strongly vary with device fabrication, the isotope effect is not sensitive to these factors. Chemicurrent experiments show a large isotope effect for H/D adsorption on surfaces.^52,71,161,162^ Thereby, they directly probe the electronic excitation while inelastic scattering experiments probe the interplay between electronic and nuclear excitation. To be able to compare our results to chemicurrent experiments, we extended our theoretical model to Ag(111) and included in it a means to predict the chemicurrent.^160^ Comparing the isotope effect between the two experiments and the theoretical model gives additional valuable insights into the adsorption process and further validates the model.

**Figure 4:**
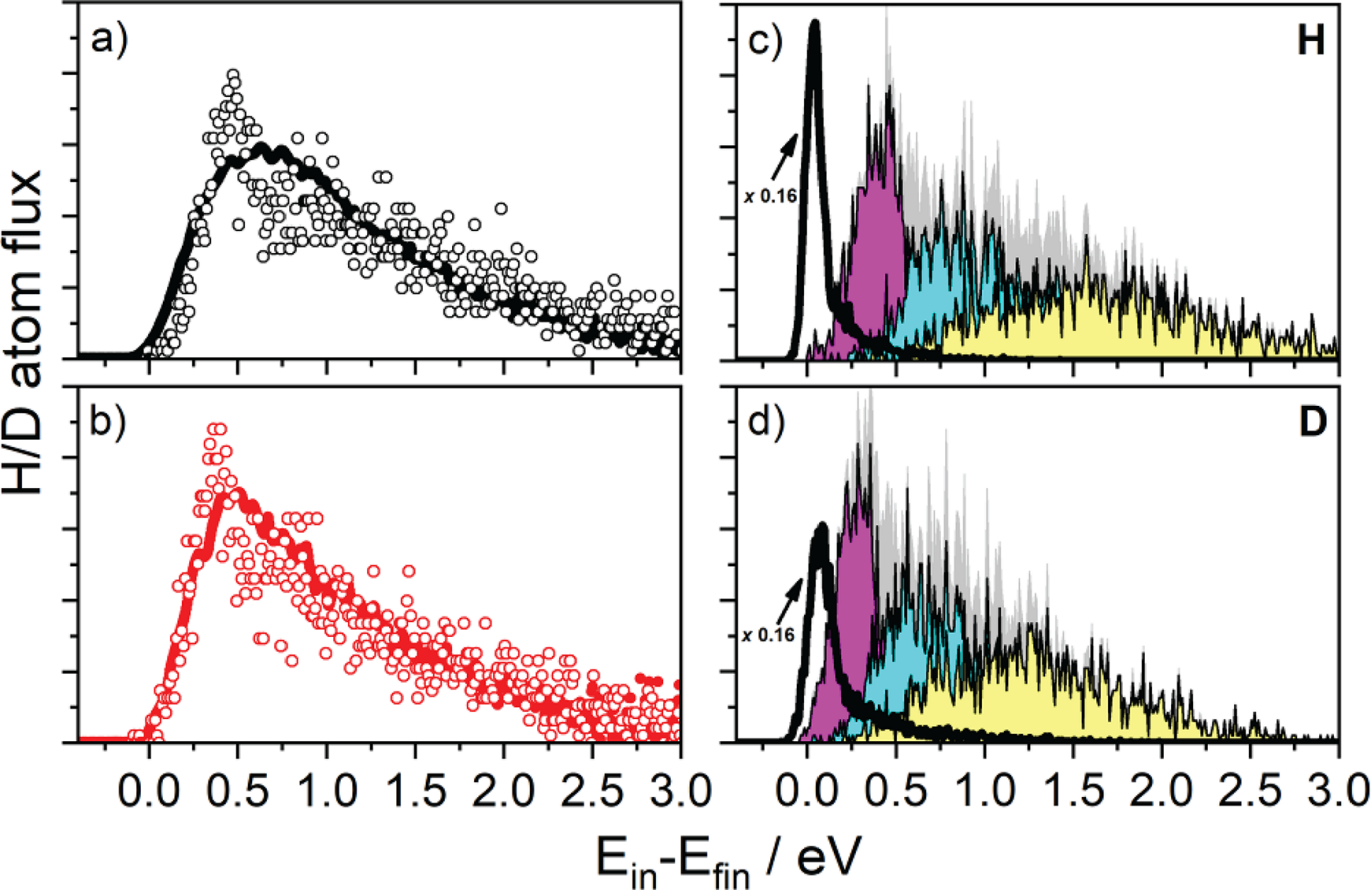
H/D isotope effect in scattering from Au(111). (a, b) Energy loss distributions for H (black) and D (red) scattering from Au(111) obtained with MD calculations (open circles) and with experiment (solid lines). (c, d) Analysis of the MD trajectories. The solid black line shows the translational energy loss probability distributions resulting from H/D collisions with Au(111) when electronic friction is not present in the model. The gray shaded area shows the probability distribution found when electronic friction is included. This distribution is divided into three classes of trajectories: single-bounce (magenta), double-bounce (blue), and more than two-bounce (yellow) collisions between H/D and a Au atom. The experimental conditions are *E*_H,in_ = 3.33 eV, *E*_D,in_ = 3.27 eV, *ϑ*_in_ = 45°, *ϑ*_s_ = 45°, *φ*_in_ = 0°, and *T*_S_ = 295 K. Adapted with permission from ref (160). Copyright 2018 National Academy of Sciences.

Typical energy loss spectra for H and D scattered from Au(111) are shown in Figure 4, parts a and b. The spectra only deviate for low energy losses but otherwise are nearly identical. We studied the dependence of the average energy loss on the incidence energy. The isotope effect Δ*E*_*H*_/Δ*E*_*D*_ is close to 1, independent of energy.^160^ The simulation shows good agreement with the experiment (compare open circles to the solid lines in Figure 4, parts a and b).

The small isotope effect in inelastic scattering results from a compensation between an increased loss to lattice vibrations and a decreased loss to electron–hole-pair excitations for D compared to H. Looking more closely at parts a and b of Figure 4, at large energy losses, the distributions are nearly identical for H and D, but for small losses (<800 meV) the D atom flux is higher. The spectra obtained from the theoretical model also show this difference, but much less pronounced than in the experiment. Furthermore, theory resembles the experimentally observed shape better in the case of D. In this energy range, scattering occurs mainly to single bounce events. For the H atom scattering, the model overestimates the importance of single-bounce events, an error that might be introduced by the classical treatment of nuclear motion. Alternatively, the long-range part of the PES might be less accurate than required. Clarifying the deviations is the subject of a current study incorporating nuclear quantum effects in the simulations.

Despite this minor issue, the theoretical simulations allow us to separate the phononic from the electronic part of the energy dissipation to the surface; see Figure 3b. It also allows us to sort the trajectories according to the number of bounces the H atom experienced with the metal surface. According to the simulations, most of the dissipated energy goes into electronic excitation in both cases (90% for H; 79% for D), but the average number of bounces is considerably larger than 1 in both cases. To experimentally resolve the phononic and electronic contributions from one another, measurements were performed with H and D at the same incidence speed.^136^ Here, the electronic coupling should be equal for H and D, but the phononic excitation is not. With this simple approach, we found from experiment that 89% of the incidence energy is transferred to electron–hole pair excitation in case of H and 68% in case of D, close to the values predicted by the theoretical simulation.^136^

A direct comparison between inelastic scattering and chemicurrent experiments is not possible. However, we were able to extend the theoretical model to describe simultaneously both the scattering experiments and the isotope effect in chemicurrent experiments. We extended the LDFA theory to describe the energy spectrum of the excited EHPs produced by the MD trajectories by implementing a forced oscillator model (FOM). From reported barrier heights and barrier transmission probabilities of chemicurrent devices, we then determined the fraction of excited electrons that can be detected as a chemicurrent. Not only is the absolute value of the predicted chemicurrent in good agreement with experiment, but so too is the isotopic ratio of chemicurrents.^160^ This helps us to understand that the large isotope effect seen for chemicurrents—while not for translational inelasticity—results from the fact that the chemicurrent is only sensitive to the electronic excitation. H atoms generate electrons with higher energies than do D atoms, further amplifying the effect. The simulations also reveal that only trajectories leading to adsorption lead to an observable chemicurrent. It would be valuable if this purely theoretical prediction could be verified experimentally in the future.

### Universal Behavior and Electronic Friction: Comparing Metals

The observations and conclusions presented above appear to be generally applicable to other metals.^158^ We carried out scattering experiments like those described above for six *fcc* transition metals: Ni, Cu, Pd, Ag, Pt, and Au, all with the (111) facet. The goal was to understand the influence of the metals’ mass and electronic structure on the energy loss. Since the metals belong either to group 10 or group 11 (coinage metals) of the periodic table, the mass changes as one moves vertically within periodic table (Cu to Ag to Au or Ni to Pd to Pt) and the electronic structure changes as one moves horizontally (Ni to Cu, Pd to Ag, or Pt to Au). Two interesting electronic properties of the metals that one might well postulate to influence the energy transfer are the work function and the density of electronic states at the Fermi level.

**Figure 5:**
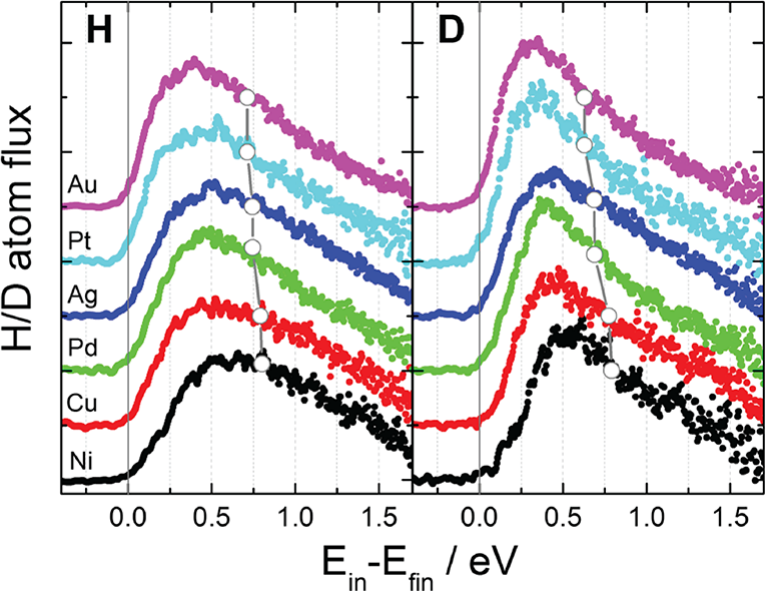
Energy loss spectra for H/D atoms scattered from six *fcc* transition metal (111) surfaces. The experimental conditions are *E*_H,in_ = 1.92 eV, *E*_D,in_ = 1.87 eV, *ϑ*_in_ = 45°, *ϑ*_s_ = 45°, *φ*_in_ = 0, and *T*_S_ = 295 K. The distributions are normalized to the area. Open circles represent the average energy loss. Reprinted with permission from ref (158). Copyright 2018 AIP.

Figure 5 shows representative results obtained for all 12 experiments, which at first glance appear nearly identical. A broad structure-less energy loss distribution with large average energy loss is seen in all cases. A closer look reveals a clear trend—energy loss decreases with increasing mass of the metal. The differences in electronic structure of the metals, by contrast, has no apparent influence on the energy loss.

As before, we constructed PESs for H atom interactions with all six metals,^139^ and the experiments were simulated with molecular dynamics using electronic friction. Again, we find uniformly good agreement between experiment and theory—slightly better agreement for D scattering than for H.

**Figure 6:**
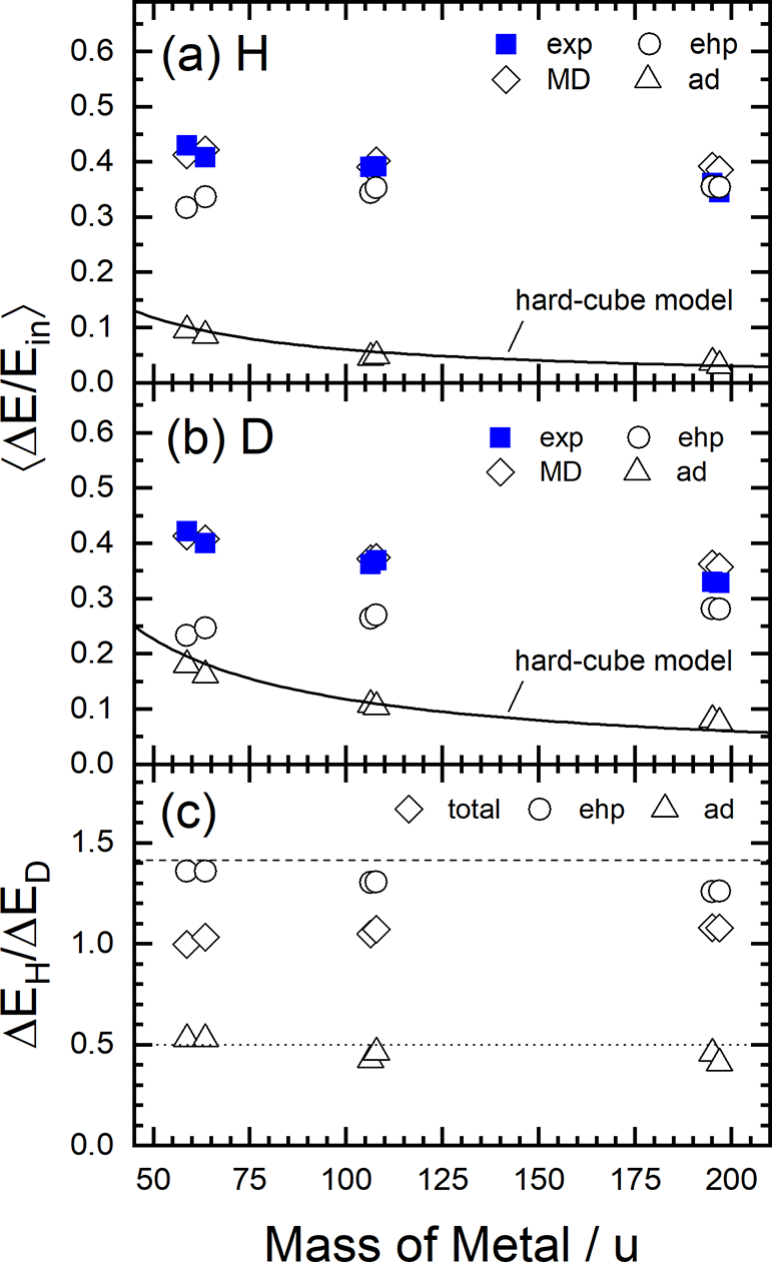
Comparison of the H atom (a) and D atom (b) average relative energy loss averaged for six metals and three incidence energies: experiment (filled squares) and EMT-LDFA-MD simulations (open diamonds). The values for the three incidence energies are averaged together. Theoretically calculated energy losses are decomposed into adiabatic (open triangles) and nonadiabatic (open circles) contributions. Solid lines show predictions of a hard-cube model. Panel c shows the dependence of the isotope effect on the mass of the surface atoms for the translational energy losses: total (open diamonds), adiabatic (open triangles), and nonadiabatic (open circles). The lines show predictions for the isotope effect of the adiabatic (dotted) and nonadiabatic (dashed) contribution of the energy loss. The scattering conditions were *ϑ*_in_ = 45°, *ϑ*_s_ = 45°, *φ*_in_ = 0, and *T*_S_ = 295 K. Reprinted with permission from ref (158). Copyright 2018 AIP.

The accurate theoretical simulations of experiment provide deeper insights into the energy loss mechanism comparing experiment to theory for all six metals at three incidence energies. We calculated the average energy loss for metal and isotope and decomposed the energy loss into contributions from electronically adiabatic and nonadiabatic contributions. Figure 6 summarizes these results showing average relative energy loss, ⟨Δ*E*/*E*_in_⟩. Panel a shows ⟨Δ*E*/*E*_in_⟩ for H and panel b that for D with all six metals. The experimental results are reproduced by the theory, exhibiting only a weak dependence on metal. The electronically adiabatic contribution decreases with increasing mass of the metal and scales like the predictions of a hard-cube model. Contrasting this behavior, the electronically nonadiabatic contribution increases somewhat with the mass of the metal. The effects tend to compensate and only a weak dependence on metal is seen. Panel c shows the isotope effect, illustrating again the compensation between adiabatic and nonadiabatic contributions to the translational inelasticity.

In summary, experiment and theory agree well for 12 metal–isotope combinations and for different incidence energies between 1 and 3 eV. The average relative energy loss is remarkably similar for all six metals, which is explained by the compensation of nuclear and electronic contributions to the energy losses. While differences in the energy dissipation to the lattice vibrations can be attributed to mass differences between the metals, the energy dissipation into the electron–hole pairs almost completely overwhelms this dependence and partially compensates it so that, in total, the energy loss is nearly independent of the metal. The absence of an influence of work function of the metal suggests that no charge transfer occurs between the surface and atom. Furthermore, neither the density of states at the Fermi level nor the character of the occupied and unoccupied surface orbitals seem to have a notable impact on the energy loss mechanism. Instead, the energy transferred is dictated by electronic friction, which depends only on the electron density, a property that is similar for the metals studied here.

All of the data presented in this paper were taken with a single incidence and scattering angle, both angles 45° from the normal to the surface designed to observe specular scattering. We also performed a detailed study of the influence of the experimental incidence conditions for H scattering from Au(111).^136^ We explored a large range of kinetic energies, incidence angles from 15° to 60°, and scattering angles from −10° to +75°. Furthermore, the influence of the azimuthal surface orientation was studied. For the other five metals, we did spot-checking of experimental parameters to confirm that similar behavior is observed as is seen for Au(111). We observe broad angular and kinetic energy distributions under all experimental conditions. We could observe no influence of the azimuthal angle of the crystal on the inelastic scattering and find a uniformly small isotope effect.^136^ All of these observations could be reproduced by our molecular dynamics models.

This encouraged us to generate a simple analytic model for H and D sticking on all six metals. We used the theory to predict the sticking probability of H and D atoms as a function of incidence angles and energy and to fit the numerically calculated sticking probabilities to a simple function describing the sticking probability’s dependence on incidence energy, incidence angle, and metal atom mass:^158,159^
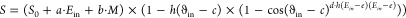
3Here *h* is the Heaviside step function, *S*_0_ = 1.081, *a* = −0.125 eV^–1^, *b* = −8.40 × 10^–4^ au^–1^, *c* = 28.88°, *d* = 0.443 eV^–1^, and *e* = 1.166 eV for H and *S*_0_ = 1.120, *a* = −0.124 eV^–1^, *b* = −1.20 × 10^–3^ au^–1^, *c* = 28.62°, *d* = 0.474 eV^–1^, and *e* = 1.196 eV for D. Note that *S*_0_ is a fitting parameter and is not related to the initial sticking coefficient that is often denoted by the same symbol in the literature.

This appears to us to be a universal empirical formula, based on first-principles analysis and verified by experiment, for accurate prediction of sticking probabilities of H or D on any metal. An important caveat here is that all experiments were carried out at room temperature—this formula has not been tested at higher temperatures, although it easily could be.

## 5 Transient Bond Formation in Collisions of H on Graphene

In this section, we present investigations of the interaction of H atoms with the 2D material graphene. When the H atom collides with a graphene surface, it is possible that electronic rehybridization occurs and a transient chemical bond forms. The bond energy that is released leads to an enormous energy initially localized in the newly formed bond. This transient bond is intrinsically unstable with respect to redissociation; but energy flow from the newly formed bond to the rest of the molecule can delay redissociation facilitating adsorption. Scattering experiments have the potential to probe directly the transient energized bond. The scattered flux exhibits a speed and angular distribution that can be understood through comparison to theoretical simulations, revealing atomic-scale processes taking place on an ultrafast time scale.

**Figure 7:**
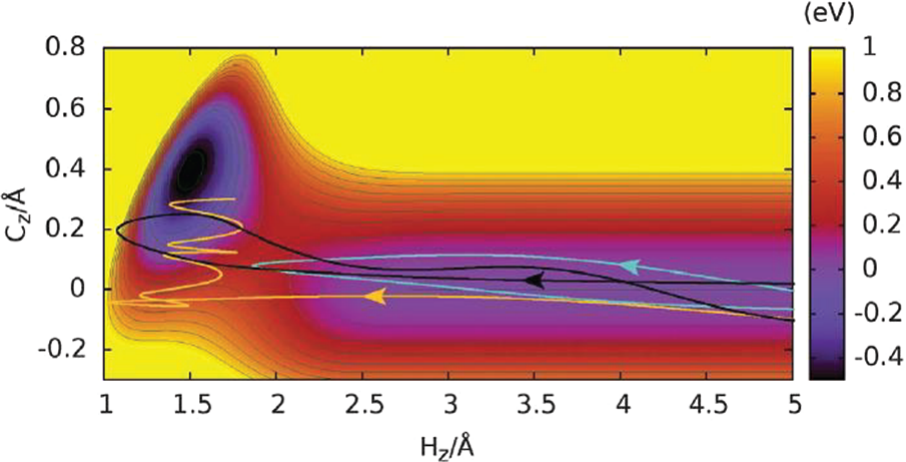
sp^2^–sp^3^ rehybridization associated with C–H bond formation during H atom collisions at a graphene surface. *H*_Z_ and *C*_Z_ are the distances of the H and C atoms from the graphene plane. One C atom must pucker out of the graphene plane to form the bond. A barrier to bond formation is clearly seen. Three example trajectories are shown: (cyan) reflection from the adsorption barrier, (black) crossing and recrossing of barrier, and (gold) adsorption. Reprinted with permission from ref (133). Copyright 2020 AAAS.

H atom scattering from graphene is quite interesting within this context.^133^ For a C–H bond to be formed, the delocalized electronic structure of graphene has to be locally destroyed, giving rise to an adsorption barrier. Figure 7 shows a 2D cut through the potential energy surface used in our work.^133^ On the *x*-axis, the distance of an H atom to the graphene surface is shown for normal incidence above a C atom top site. The *y*-axis shows the distance of the corresponding C atom to the graphene plane. For the chemisorption well to be formed, not only it is necessary for the H atom to approach the surface but also the C atom must pucker out of the surface plane. For low incidence energies, the H atom is not able to overcome the barrier and is reflected (cyan trajectory). For energies exceeding the barrier, a transient C–H bond may form, and the H atom may recross the barrier (black trajectory). Finally, it is possible for the H atom to be trapped in the chemisorption well (gold trajectory). In our experiment, we observe the outcome of the cyan and black trajectories by measuring the kinetic energy and direction of the scattered H atoms. The two cases lead to very different scattering dynamics, enabling us to follow the C–H bond formation and the energy flow from the bond to the graphene layer.

### The Mechanism of H Atom Adsorption

Ideally, experiments on free-standing graphene could be compared to theory most simply. Unfortunately, this is not yet practical. Instead, we grew graphene on a Pt(111) substrate, which is a system where graphene interacts only weakly with the substrate by van der Waals forces—it is not free-standing graphene, but it is close.^163^ A further complication results from the several rotational domains of graphene found in these samples.^163,164^ This has to be considered when comparing to theoretical calculations. Experimentally, graphene was grown *in situ* on platinum by exposure to ethylene at elevated temperatures.

**Figure 8:**
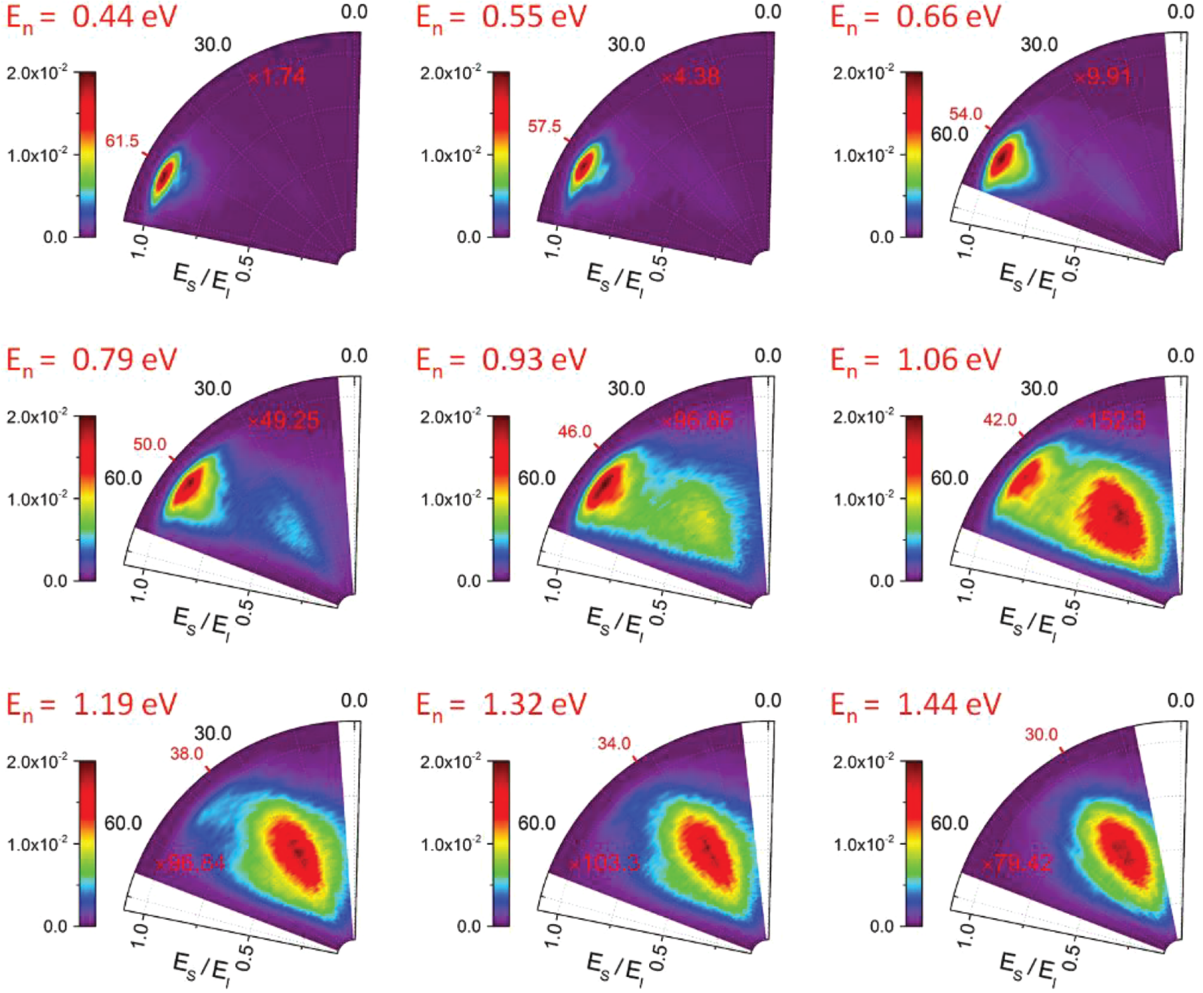
Angle and energy resolved scattering flux for H atoms incident with *E*_I_ = 1.92 eV on a graphene surface grown on Pt(111). The scattered energy *E*_S_ is shown as a fraction of *E*_I_ along the radial axis and the scattering angle, *ϑ*_*S*_, is shown along the polar axis. By changing the incidence angle, *ϑ*_in_, the normal component of H atom energy, *E*_n_, is varied. For low *E*_n_, only quasi-elastic scattering is observed. Increasing *E*_n_ results in a second channel with high energy loss that eventually dominates at large *E*_n_. The red tick marks the specular angle. The integrated intensity for one experimental condition (*E*_I_ = 0.99 eV, *ϑ*_in_ = 63.5°, not shown) was normalized to 1, and the integrated intensity of all other distributions is shown relative to that distribution. Each distribution is multiplied by a factor indicated in red.

**Figure 9:**
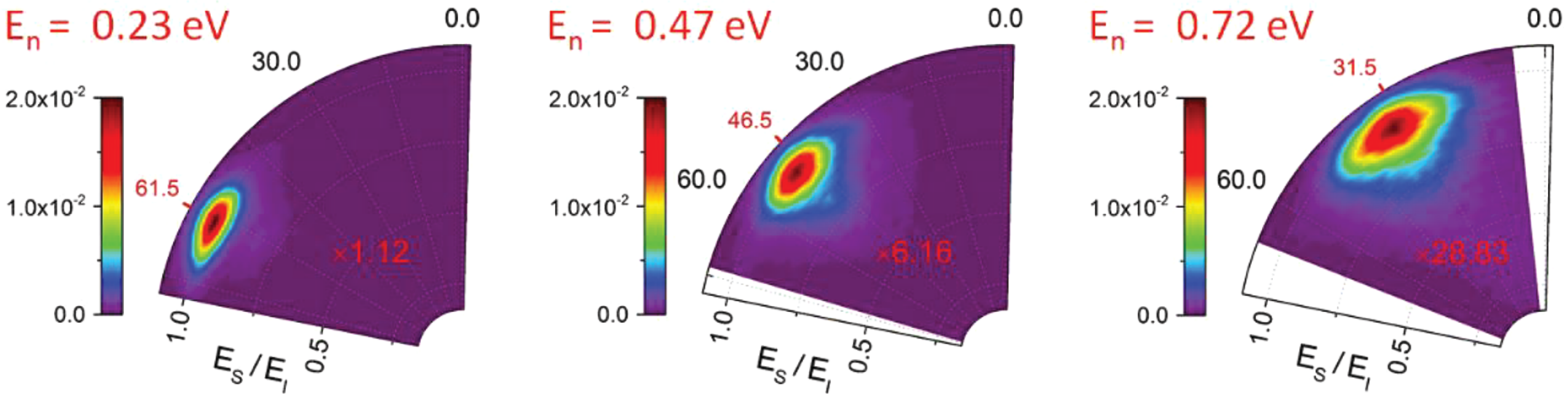
Angular and energy resolved scattering flux for H atoms incident with *E*_I_ = 0.99 eV on a graphene surface grown on Pt(111). By changing *ϑ*_in_, *E*_n_ is varied. For low *E*_n_, only quasi-elastic scattering is observed. Increasing *E*_n_ results in a drop in intensity as H atoms adsorb with increased likelihood. Otherwise, this figure is as in Figure 8.

Figures 8 and 9 show angle-resolved energy distributions of H atoms scattered from graphene on Pt(111) for two different incidence energies, *E*_I_ = 1.92 and 0.99 eV, respectively. The radius of the polar plots denotes the relative energy loss and the angle corresponds to the scattering angle. A red tick marks the specular angle. As the incidence (specular) angle, *ϑ*_in_, decreases, the normal component of incidence energy, *E*_n_, increases. Only the normal component of incidence energy is useful to cross the barrier to C–H bond formation. Therefore, the crossing probability increases as the incidence angle decreases. Let us look at this in detail.

For *E*_I_ = 1.92 eV incidence energy and 61.5° incidence angle (Figure 8 top left), the normal energy is *E*_n_ = 0.44 eV, barely enough to overcome the barrier. Most of the H atoms are directly scattered before passing over the barrier exhibiting nearly elastic, nearly specular scattering. As *ϑ*_in_ decreases, *E*_n_ increases, and a higher proportion of H atoms cross the barrier, forming a transient C–H bond. In the scattering distributions, the fast component decreases and a new component with high energy loss (∼1 eV) and lower scattering angle emerges. For *E*_n_ above 1 eV, the slow component dominates the spectrum. Similar behavior is observed in Figure 9 for the *E*_I_ = 0.99 eV data. The fast component decreases with increasing *E*_n_; however, the slow component is absent. This is due to the fact that the H atoms that cross the barrier do not have enough excess energy to recross—instead they adsorb to the graphene surface.

We developed a theoretical model to describe the graphene scattering in collaboration with Miller and co-workers.^133^ A reactive empirical bond order potential (REBO)^165^ was fitted to electronic structure data obtained by embedded mean-field theory (EMFT).^166^ The resulting PES was used to perform both classical molecular dynamics simulations and approximate quantum mechanical ring–polymer molecular dynamics.^89^ The effect of the Pt substrate was modeled using Lennard-Jones potential interactions with each atom in the graphene layer.^133^ In agreement with the experiment, the simulation also results in a bimodal scattering distribution in which the ratio of the two channels depends on the normal energy. The best agreement with experiment is obtained when the incidence angle used in the simulation is shifted from the experimental value by 10°. We attribute this discrepancy to errors in the PES. Still, the basic features of the experiment are well described.

**Figure 10:**
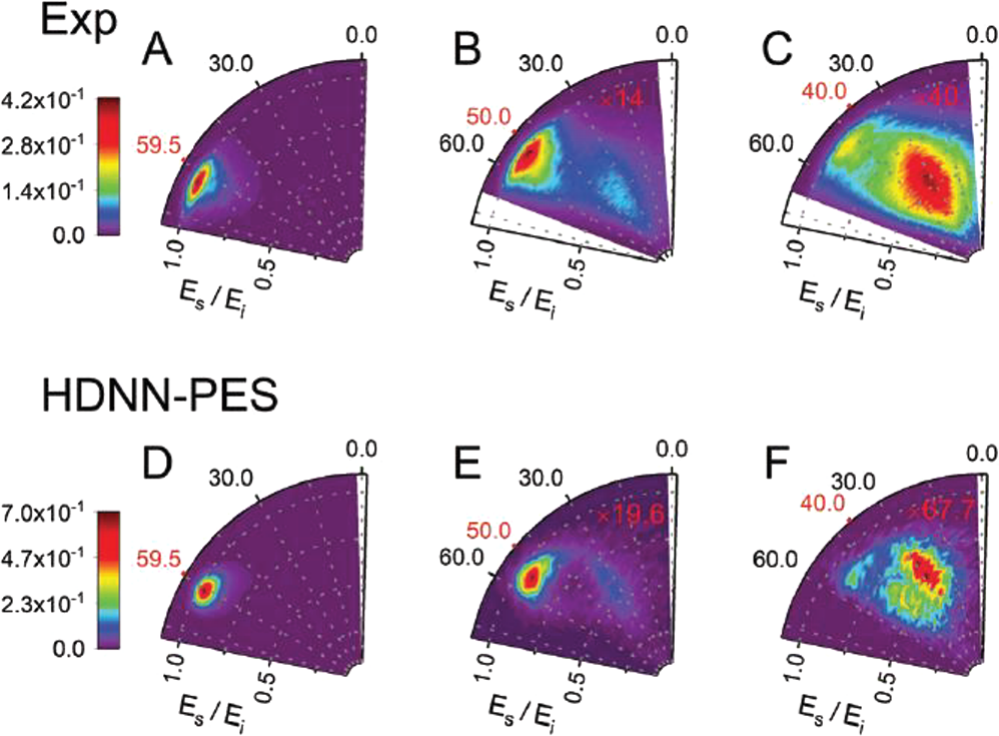
Comparison of experimentally obtained and theoretically calculated angle and energy resolved scattering flux for H atoms incident with *E*_I_ = 1.92 eV on graphene: (A–C) experimental; (D–F) theoretical, where each distribution is based on one million trajectories. Otherwise this figure is as in Figure 8. Adapted with permission from ref (145). Copyright 2021 PCCP Owner Societies.

Recently, we developed a new PES using neural networks,^145^ considerably improving the quality of the PES. Figure 10 shows a comparison of molecular dynamics simulation using the new PES in comparison with experiment. Here, the effect of the substrate is not included, suggesting that only a minor substrate effect is present in case of platinum. Theory verifies the hypothesis that the fast component is due to atoms scattered from the barrier. The slow channel is due to H atoms that get much closer to the graphene layer. Interestingly, nearly all of the scattered atoms underwent only a single collision, even the ones that formed a transient C–H bond. The corresponding interaction time is very short (only around 10 fs), resulting in a remarkably rapid energy transfer of up to 1 eV in 10 fs.

### The Sticking Probability versus *E*_I_: Benchmark for Theory

While it is straightforward to extract sticking probabilities from the theoretical simulations, it is difficult experimentally. Since we only measure in the plane perpendicular to the tagging lasers and the incident beam, loss of H atom flux can be due to adsorption or to out-of-plane scattering. Experimentally, we can turn the azimuthal angle of the crystal and record the influence on the in-plane scattering signal. If the signal does not depend on the azimuthal angle, a cylindrically symmetric scattering distribution can be assumed. For the fast (quasielastic) component, this assumption was verified to be correct; however, for the slow component, it is not. Transient C–H bond formation introduces directional forces that cause the plane of scattering to be rotated with respect to the surface normal. For this reason, we restricted out analysis of sticking probabilities to the 0.99 eV data, which exhibits no slow component.

**Figure 11:**
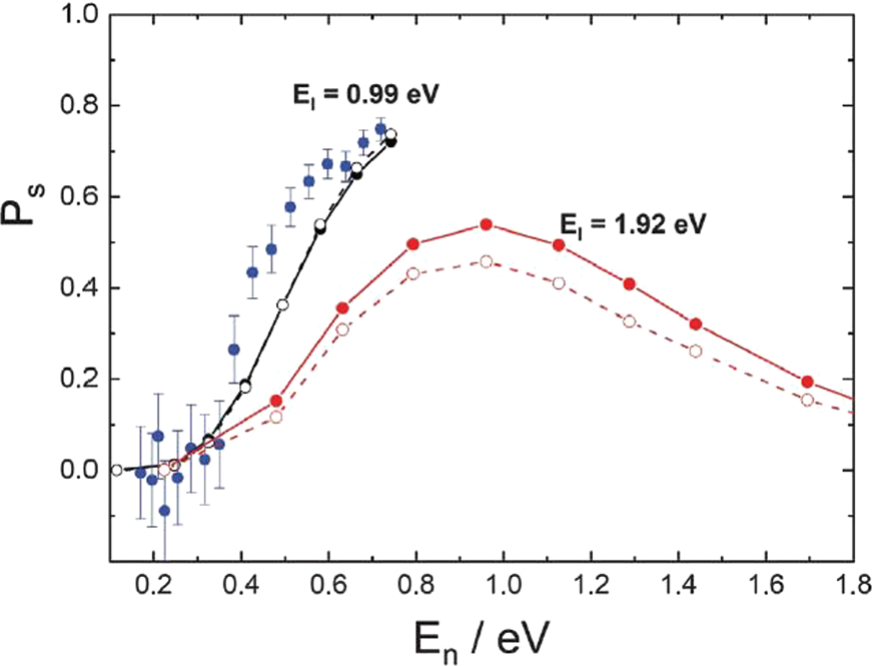
H atom sticking probabilities at graphene as a function of *E*_*n*_. Experimentally derived (blue) and theoretically predicted (black) sticking probabilities for *E*_I_ = 0.99 eV plotted against the normal component of the incidence energy (*E*_n_). Theoretically predicted sticking probabilities for *E*_I_ = 1.92 eV are shown in red. Theoretical simulations used a full dimensional EMFT-REBO PES that includes the influence of the Pt substrate with classical molecular dynamics (solid symbols) or ring polymer molecular dynamics (open symbols). Reprinted with permission from ref (133). Copyright 2020 AAAS.

Figure 11 shows the experimentally derived sticking probabilities for *E*_I_ = 0.99 eV as a function of *E*_n_. Theoretically derived sticking probabilities are shown as circles without error bars. Results of classical molecular dynamics simulations are shown with filled symbols, and those obtained with ring polymer molecular dynamics are shown with open symbols. Theoretical predictions are shown for both incidence energies. For *E*_I_ = 0.99 eV, the agreement of the theoretical simulations with experiment is good. It is interesting to note that there is very little predicted nuclear quantum effect, as one might expect tunneling of H atoms to play an important role. Theory allows us to predict sticking probabilities for the 1.92 eV H atoms. Here, a maximum in the sticking is found for a normal energy of ∼1 eV, indicating that the normal energy is also effective for recrossing of the barrier.

Good agreement between experiment and theory justifies using the theoretical simulation to take a closer look at the dynamics of the energy transfer process. The first conclusion we can draw from our observations is that much of the energy loss and adsorption can be explained within the BOA—the adiabatic model gives a good description of experiment. The theoretical model reveals that in an interaction time of only 10 fs an energy transfer of more than 1 eV takes place. The collective motion of the carbon framework thereby takes up the energy. When the transient C–H bond is formed, the system is far from its equilibrium: graphene itself is flat; however, the equilibrium structure after a C–H bond is formed is characterized by one C atom puckered out of the plane and C–C bonds changing length dramatically. When the H atom approaches the graphene layer, the carbon atoms start to move and the carbon–carbon bonds to the carbon forming the transient C–H bond want to extend resulting in an initial in-plane motion of the neighboring atoms. However, the surrounding carbon hinders the in-plane motion and the central carbon atom starts to pucker out of the surface. Before this can happen, the H atom has already begun to leave the surface, but the energy required to initially deform the graphene layer cannot be transferred back to the translational motion of the H atom. In other words, when the transient C–H bond is formed, the energy is rapidly distributed to many normal modes of the system, and only a portion of the energy can go back to the H atom when the bond breaks again.

Here, one very important difference to femtosecond pump–probe experiments studying energy flow out of a bond becomes apparent. When a new bond is formed in a chemical reaction, the system can be far away from its equilibrium configuration. Such a configuration can rarely if ever be directly prepared by absorption of a photon. The initial positions of the nuclei will have a profound influence on the internal vibrational energy redistribution that stabilizes the newly formed bond. Scattering experiments make such configurations accessible and with the support of a theoretical model can give very detailed insights into the dynamics of new bond formation.

Graphene is a very interesting model system, providing an opportunity to study the formation of a chemical bond in detail. We were able to follow the energy flow out of a newly formed bond into the graphene and understand the adsorption process. While qualitative agreement is already achieved between experiment and theory, quantitative agreement is not yet satisfying, motivating further studies.

## 6 Perspectives for the Future

We have shown that the use of photolytic atom beams in combination with Rydberg atom tagging TOF enables interesting inelastic surface scattering experiments, which have the particular advantage of providing good data for comparison to theory. Before finishing this paper, we would also like to provide some ideas about future direction for research made possible by this method.

All of the inelastic scattering results presented above relied on an excimer laser for hydrogen halide dissociation. Much can be accomplished by using dye lasers for photolysis, expanding the range of H atom incidence energies. Higher energies can be achieved by utilizing predissociating Rydberg states accessed by four wave mixing of two dye laser beams to produce VUV.^167^ Energies up to 7 eV have been demonstrated, allowing possible detection of H atom collision induced electron emission or anion (H^–^) formation. Observation of the dynamical signatures of such processes would provide interesting new data for development of theories of surface electron emission and electron transfer. VUV photolysis is also a way to produce remarkably low incidence energies with extremely narrow velocity spreads. This results from the fact that VUV photolysis of HX can produce X in highly excited electronic states producing low energy H atoms.^168^ Such beams are good candidates for experiments designed to observe quantum resonances and diffraction of H atoms scattered from a surface.

We are also now able to begin work with an improved sample mount and vacuum system to perform experiments at surface temperatures <10 K. This has required substantial improvements to the apparatus as, at such low temperature, surface contamination is harder to avoid. We note that the H atom scattering signal is very sensitive to surface contamination—we observed that a base pressure of 10^–10^ mbar is not sufficient for many experiments when the surface temperatures are below 200 K. Low surface temperatures are interesting for two main reasons. First, we want to push the resolution of the experiment to the limit by doing scattering experiments where the thermal motion of the surface atoms can be minimized. This might allow us to test the theoretical prediction that the broad energy loss distribution observed at room temperature for metals resolves itself into multiple separate features at low surface temperatures.^169^ The second reason to pursue this is that quantum effects are in general more important at low temperature, and high dimensional quantum surface scattering theories are in desperate need of benchmark data. Low temperatures also invite experiments on superconducting materials.

A wide variety of new experiments are also possible by exploring different solids and surfaces. We want to extend our work on metals beyond the *fcc* metals and (111) surface facets highlighted in this review. Furthermore, we will extend our experiments to semiconductors and insulator surfaces. This will be made easier by a newly installed load lock sample transfer system, which allows samples to be exchanged on a daily basis.

There are other interesting directions to pursue based on properties of photolytic atom sources. Using circularly polarized light, a spin-polarized H atom beam can be produced.^170−173^ Combining this source with spin-selective detection, scattering with magnetic surfaces could be studied. Pump–probe style experiments with ultrashort (∼100 ps) H atom pulses is another idea that emerged in our laboratory some years ago.^174^ A new machine based on this idea is now operating in Göttingen, with which one can study H atom scattering from laser-excited surfaces. Finally, photolysis can be extended to other atoms of the periodic table—C, O, and N atoms—by photolysis of diatomic molecules like O_2_, NO, and CO using VUV light from a free electron laser. As atoms produced in different electronic states travel with different velocities from the photolysis region, they arrive at different times at the surface. Hence, the prospect of electronically state selected atom beams is also near.

From the point of view of theory, there are two directions deserving immediate attention in the future. First, the HDNN-PES was constructed for the H atom interacting with freestanding graphene, whereas in the experimental one, one has to use a substrate (Pt, Ni etc.). One can certainly apply a similar approach to develop an accurate HDNN-PES accounting for the substrate to study to which extent various substrates influence hydrogen scattering dynamics from graphene. Possible experiments to aid in this involve changing the substrate from the weakly interacting Pt(111) to the strongly interacting Ni(111).^175,176^ This will give valuable information about the magnitude of the substrate effect. We may also use highly oriented pyrolytic graphene (HOPG) as “graphene on graphite”. Beyond this, the question on the importance of quantum-mechanical effects in the hydrogen scattering and adsorption remains open. Utilizing modern effective quantum-mechanical propagation algorithms would shed a light on this topic. We consider the multi-configuration time dependent Hartree approach as one of the most promising in that respect, since reliable simulations are possible for systems with dozens degrees of freedom.
